# The effect of a self-learned virtual learning package on knowledge, attitude, and self-care behaviors of COVID-19 in people referred to health and treatment centers

**DOI:** 10.1186/s12889-024-19233-y

**Published:** 2024-06-26

**Authors:** Tayebeh Rakhshani, Seyyed Manoochehr Dolatkhah, Seyyed Mansour Kashfi, Ali Khani Jeihooni

**Affiliations:** 1 Nutrition Research Center, Department of Public Health, School of Health, Shiraz University of Medical Sciences, Shiraz, Iran; 2 Department of Public Health, School of Health, Shiraz University of Medical Sciences, Shiraz, Iran

**Keywords:** Educational intervention, Knowledge, Attitude, Self-care behaviors, COVID-19

## Abstract

**Background:**

COVID-19 is one of the most common diseases in recent years, the most important way to prevent is through self-care behaviors; therefore, it is important to these behaviors in people. According to the importance of promoting self-care behaviors of this disease, and according to the characteristics and effectiveness of interventions based on behavior change, this study aimed to investigate the effect of educational intervention on self-care behaviors of COVID-19 in a group of patients.

**Methods:**

This quasi-experimental study was conducted on 164 people who referred to health and treatment centers in Dehdasht City, Iran. The cluster sampling method divided the participants into experimental and control groups at random (82 people for each group). Data collection tool was a researcher-made questionnaire completed by the control and experimental groups before and three months after the intervention. The intervention program in this training group is to form a WhatsApp group and send messages in the form of audio files, text messages, text messages with photos, video messages, and PowerPoints. After creating the group and adding the participants, according to the agreement with the group members, every day of the week (8:00 am to 12:00 pm) to send educational files through the WhatsApp application. Also, the group members could ask their questions and problems to the researcher during the designated hours. The control group was also given routine care and follow-up at the centers, and no training was given regarding self-care behaviors. After entering the SPSS 24, data were analyzed by independent t, chi-square, and paired t statistical tests.

**Results:**

164 individuals working in healthcare services from health and treatment centers were included in this study. Before the intervention, demographic characteristics such as marital status, education level, medical history, and smoking history were similar between the two groups (*P* > 0.05), as indicated by the results of chi-square tests. Furthermore, there were no significant differences in the mean scores of knowledge, attitude, and self-care behaviors between the experimental and control groups prior to the intervention (*P* > 0.05), according to independent t-tests. Following the intervention, notable changes were observed. The post-intervention analysis revealed statistically significant differences between the experimental and control groups in terms of knowledge, attitude, and self-care behaviors (*P* = 0.001). Specifically, the experimental group exhibited significant improvements in these variables compared to the control group.

**Conclusion:**

In this study, education led to the improvement of self-care behaviors in people who referred to health centers. Considering the importance of the role of health education in promoting self-care behaviors as well as preventing infectious diseases such as COVID-19, it is suggested that educational interventions focus on self-care behaviors in other diseases.

## Background

One of the most fatal respiratory infectious diseases that recently became a concern is COVID-19, the latest coronavirus, which was detected in Wuhan, China, in late 2019. China reported the first death from this virus on January 11. And in a short period of time, it spread to most countries in the world and affected more than 18 million people, resulting in the deaths of more than 700,000 of the world’s employees [1–3]. Until March 1, 2020, 67 countries in the world were involved in this disease, and 88,340 positive cases of this disease were reported throughout the world, of which 3,001 deaths and 42,728 cases were recovered. Of these, the first 12 countries that conflicted with this disease included China, South Korea, Italy, Iran, Japan, France, Germany, Singapore, Hong Kong, Spain, America, and Bahrain, respectively [4].

This disease spread rapidly in Iran, and according to the report of Iran’s Ministry of Health, Treatment, and Medical Education in June 2020, 202,584 confirmed cases of COVID-19 and 9,507 deaths from this disease were recorded in the country [5]. Iran was the 10th country with 257,303 infected cases until July 2019. In Iran, until July 2019, the number of people who died due to the COVID-19 infection was 12,829, and the number of people infected with this disease until April 2019 was 1309 in Mashhad, 252 in Neishabor, 160 in Sabzevar, 340 in Torbet Heydarieh, and 70 in Gonabad [6, 7].

In addition to the widespread prevalence of this disease and the lack of treatment for it at that time, one of the most important factors for preventing this disease was the self-care behaviors of people, which were based on knowledge and attitude towards these behaviors [8]. Experiences in this regard have shown that taking preventive measures in order to control the disease requires social awareness from all people, and one of the most important measures is to determine the level of knowledge, attitude, and self-care behavior and improve these components [9–12]. The results of some studies have shown that a high level of knowledge about a disease is directly related to self-care behaviors [13–16], and some other studies have shown that a high level of attitude can predict these behaviors [17–20].

In fact, self-care is a series of actions that are performed voluntarily and without coercion by people in society to create, maintain, and improve their level of health [21]. These behaviors could lead to people’s adaptation to life’s stresses and crises and increasing independence in performing daily activities [22]. Therefore, due to the importance of considering self-care behaviors, preventive measures such as education and raising the level of attitudes and preventive skills for personal protection against this disease were one of the most important and necessary strategies of the program for the prevention and control of COVID-19 [23].

Self-preventive measures encompass a range of actions aimed at minimizing the risk of contracting and transmitting the virus. These behaviors typically include practicing good hand hygiene by washing hands frequently with soap and water for at least 20 s, or using hand sanitizer with at least 60% alcohol when soap and water are not readily available. Additionally, wearing face masks in public settings, maintaining physical distancing of at least 6 feet from others, and avoiding large gatherings are recommended preventive measures to reduce the risk of viral transmission. The significance of these self-preventive behaviors lies in their effectiveness in interrupting the chain of transmission of the virus, particularly in situations where specific treatments or vaccines are not readily available. Promoting awareness and understanding of these self-care behaviors is crucial for empowering individuals to take proactive steps in safeguarding their health and well-being amidst the ongoing public health crisis.

Given the lack of specific treatments available during the study period, the emphasis on self-care behaviors as a primary means of disease prevention becomes even more critical. Previous studies have indicated that individuals’ levels of knowledge, attitude, and engagement in self-care behaviors directly influence the spread and impact of infectious diseases like COVID-19 [24–26]. For example, in Shahnazi et al.‘s study, the importance of self-care behaviors in preventing COVID-19 has been mentioned [27] and in the same study by Tadesse et al., the application of self-care behaviors based on the health belief model has been mentioned in the prevention of COVID-19 [24]. In another study conducted by Khazai Yul et al., self-care behaviors and self-efficacy of people as the most important predictors of COVID-19 were introduced [25]. Therefore, investigating the efficacy of educational interventions aimed at enhancing self-care behaviors is not only timely, but also essential for guiding public health strategies and mitigating the transmission of the virus within communities. Therefore, according to the importance of promoting self-care behaviors for this disease, by focusing on a localized context in Dehdasht City, Iran, this research contributes valuable insights into the effectiveness of such interventions within specific demographic and cultural settings, by performing a self-learned virtual learning package in enhancing these behaviors.

## Methods

### Research design

The target group took part in this quasi-experimental study were the health care personnel, working in the health and treatment network of Dehdasht City, Iran, in 2021.

### Sample size and sampling

The criteria for entering the study for the test group were to refer to the health centers of Dehdasht city, to have a smartphone having the ability to install the WhatsApp social network, suitable visual and auditory performance, and the willingness to participate in the study, and the criteria for dropping out of the study were unwillingness to cooperate at any time. To determine the sample size by considering the average comparison formula in two communities and also according to the results of the study by Elgzar et al., the confidence level of 0.95%, the power of the test of 80%, and the attrition rate of 10% for each group of 82 people were calculated [28]. The sampling method was cluster sampling. In this way, from the number of centers covered by the health and treatment network of Dehdasht city, two centers were randomly selected: one as the control and one as the experimental group. The researcher, based on the household file number and considering the entry criteria, selected 82 people at random for the study.

$$n={\left(\frac{({Z}_{1-\raisebox{1ex}{$\alpha $}\!\left/ \!\raisebox{-1ex}{$2$}\right.}+{Z}_{1-\beta })}{0.5 \times ln\left(\frac{1+r}{1-r}\right)}\right)}^{2}+3$$

n: Sample size

Z_1_: Z-Score of the normal distribution

α: Type I error rate

β: Type II error rate

r: The expected correlation coefficient

### Educational intervention

#### Content development

To design the intervention, people were invited once to follow the health principles to the referring centers, and the researcher’s questionnaire was collected from both groups. Finally, the weaknesses and strengths of the participants were found through their initial assessment by filling out the questionnaires, and the intervention started for the experimental group. After reviewing relevant literature, guidelines from health organizations (such as WHO and CDC), existing educational materials, consulting with experts in the field, including a pulmonologist, psychologists, epidemiologist, and other relevant professionals the educational content was developed. The content was also developed in various formats to cater to different learning preferences and accessibility needs. Considering the cultural context of the target population in Dehdasht City, the content may have been tailored to resonate with their beliefs, values, and practices. This aspect is crucial for ensuring the content’s acceptability and effectiveness among the participants.

#### Content outline

The educational content included introduction to COVID-19 and its transmission, importance of self-care behaviors in preventing COVID-19, key self-care behaviors: hygiene practices, mask-wearing, social distancing, seeking medical help, understanding symptoms and when to seek medical attention, addressing myths and misconceptions about COVID-19, and coping strategies for managing stress and anxiety related to the pandemic.

#### WhatsApp group setup

First, a mobile phone number connected to WhatsApp social networks was received to send educational files. A WhatsApp group was created for the experimental group participants. Then the consent of the participants were obtained to join the group and receive educational materials.

#### Delivery of educational materials

This training group’s intervention program is to form a self-learned virtual learning package. The educational materials were sent through WhatsApp which included, audio files (10 recordings covering key concepts), text messages (150 messages reinforcing key points), messages with photos (5 visual aids illustrating self-care practices), video messages (3 videos demonstrating proper hygiene techniques), and PowerPoint presentations (1 slide deck summarizing essential information).

#### Interactive engagement

The participants were encouraged to engage with the content by asking questions and sharing their experiences. Moreover, support and clarification were provided during designated hours (8:00 am to 12:00 pm).

#### Routine care for control group

We ensured that the control group received routine care and follow-up at health centers. We also avoided providing specific training on self-care behaviors to the control group to maintain the study’s integrity.

Three months after the intervention, we gathered the data again and compared it to the pre-intervention period. A group of experts, including a mental health expert, an expert on the prevention of communicable diseases, and an expert on the prevention of non-communicable diseases, were used in the design of the educational content. To follow the ethical guidelines, a similar educational program was held for the control group after the completion of the study.

#### Instruments

##### Demographic information questionnaire

This survey included demographic information (marital status, education, medical history, and smoking history).

##### A researcher-made questionnaire of self-care knowledge, attitude, and behavior

This questionnaire includes three questions: knowledge (18 questions), attitude (8 questions), and behavior (5 questions).

##### Knowledge

This section contained 18 questions with a 3-point Likert scale (true, false, and don’t know) related to knowledge of respiratory infections. The lowest score received 18 points, while the highest score received 54 points.

##### Attitude

*This section comprised eight questions on a 5-point Likert scale, ranging from complete agreement to complete disagreement, addressing issues related to preventive behaviors against COVID-19. The lowest score received 8 points, while the highest score received 40 points.*

##### Behavior

*This construct included nine questions with a 2-choice scale (yes and no) regarding the perception of Gachsaran city hospital personnel regarding respiratory infection prevention behaviors.* The lowest score receives 9 points, while the highest score receives 18 points.

### The tool’s validity and reliability

Twelve experts in the field of health education and promotion, including a pulmonologist, two psychologists, and an epidemiologist, evaluated the face validity of the questionnaire. We examined the content validity using the content validity index (CVI) and content validity ratio (CVR). To compute the CVI, the board of experts measured it. The Lawshe table index eventually revealed that each item was greater than 0.56, indicating the necessity of keeping the related questions for further analysis.

We used internal consistency methods to measure the instrument’s reliability. In this study, we distributed a questionnaire among 30 eligible participants to determine the internal correlation of different parts of the instrument. After data analysis, the Cronbach’s alpha coefficient was determined for all three factors (knowledge, attitude, and behavior) and for the whole questionnaire. Cronbach’s alpha was 0.76 for knowledge structure, 0.75 for attitude structure, and 0.81 for self-care behaviors.

### Data analysis

The data was analyzed by SPSS 24. We initially assessed the normality of the data through the Kolmogorov-Smirnov test. To describe the data, frequency indicators, mean, and standard deviation were implied, and to analyze the data, independent t-tests, chi-square tests, and paired t-tests were implied. A significance level of 0.05 was considered for all tests.

## Results

164 individuals working in healthcare services from health and treatment centers were included in this study. The demographic characteristics of the participants are summarized in Table 1. The chi-square test indicated no significant differences between the experimental and control groups in terms of marital status (χ² = 0.423, df = 1, *p* = 0.423), education level (χ² = 2.278, df = 2, *p* = 0.184), medical history (χ² = 2.374, df = 1, *p* = 0.156), and smoking history (χ² = 0.599, df = 1, *p* = 0.247).

**Table 1:** Comparison of frequency distribution of demographic variables in two study groups

Variable		Experimental group		Control group		*P*-value
		Number	Percentage	Number	Percentage	
Marital status	Single	15	18.3	10	12.2	0.423
	Married	67	81.7	72	87.8	
Educational level	Undergraduate	15	18.3	13	15.8	0.184
	Graduate	25	30.5	22	26.8	
	Master’s degree or higher	42	51.2	47	57.4	
Medical history	Without medical history	25	30.5	35	42.6	0.156
	With medical history	57	69.5	47	57.4	
Smoking history	Yes	29	35.3	32	39	0.247
	No	53	64.7	50	61	

The results of the independent t-test revealed no significant differences between the experimental and control groups in the mean scores of knowledge (t = 1.539, df = 162, *p* = 0.165), attitude (t = -1.649, df = 162, *p* = 0.095), and self-care behaviors (t = -1.509, df = 162, *p* = 0.140) before the intervention.

However, after the intervention, significant differences were observed between the two groups in all three variables. Table 2 presents the comparison of the average scores of knowledge, attitude, and self-care behaviors before and three months after the intervention in both groups. Additionally, paired t-tests revealed significant improvements within the experimental group in knowledge (t = -28.195, df = 81, *p* < 0.001), attitude (t = -39.751, df = 81, *p* < 0.001), and self-care behaviors (t = -20.327, df = 81, *p* < 0.001) after the intervention.

These findings suggest that the self-learned virtual learning package effectively enhanced knowledge, attitude, and self-care behaviors related to COVID-19 among participants referred to health and treatment centers in Dehdasht City, Iran.

**Table 2:** Comparison of the average scores of knowledge, attitude, and self-care behaviors, before and 3 months after the intervention in both groups

Variable		Experimental group	Control group	*P*-value*
		M ± SD	M ± SD	
Knowledge	Before intervention	21.23 ± 1.82	19.77 ± 1.09	0.165
	3 months after intervention	32.02 ± 1.40	21.77 ± 1.42	0.001
	*P*-value**	*P* < 0.001	0.940	
Attitude	Before intervention	19.38 ± 1.03	20.17 ± 1.35	0.095
	3 months after intervention	33.19 ± 1.29	23.12 ± 1.36	0.001
	*P*-value**	*P* < 0.001	0.141	
Self-care behaviors	Before intervention	11.04 ± 1.46	11.80 ± 1.43	0.140
	3 months after intervention	15.16 ± 1.22	10.10 ± 1.17	0.001
	*P*-value**	*P* < 0.001	0.082	

*Independent t-test

**Paired t test

## Discussion

This study was conducted with the aim of determining the effect of a self-learned virtual learning package on self-care behaviors of COVID-19 in people referring to the health and treatment network in Dehdasht City. In this study, a training program based on a virtual training package using the WhatsApp application (sending text messages, photos, and training videos) was conducted for the intervention group. In these meetings, people learned about the self-care program and its effect on the prevention of COVID-19 and the importance of participating in training sessions, which improved the behavior of the prevention of this disease.

Following the intervention, there was a substantial increase in knowledge among the experimental group compared to the control group. This enhancement can be primarily attributed to the training provided through the virtual learning package. Participants gained a deeper understanding of COVID-19 and its preventive measures, likely due to the structured educational content delivered via WhatsApp. Moreover, the heightened awareness about infectious diseases, coupled with the ongoing COVID-19 pandemic, motivated participants to engage more actively in learning and adopting preventive behaviors. The knowledge gained through the intervention empowers individuals, particularly healthcare workers, to effectively educate and guide others in disease prevention strategies.

The current study’s findings demonstrated that, following the intervention, there was a statistically significant difference in attitudes between the two groups, with the experimental group’s average attitude being greater than that of the control group. This conclusion is in line with the findings of investigations conducted by Hedayati et al., Zaildo et al., Calcagni et al., and Lapoirie et al. [29–32]. In justification of this finding, it can be stated that training can make the educated person aware of the disease, and increasing knowledge can lead to an improved attitude, and a person accepts that with an increasing attitude towards infectious diseases, he can better observe self-care. They are safe from infectious diseases because they are aware of health and safety concerns.

The intervention also led to a significant improvement in attitudes towards COVID-19 among participants in the experimental group. This shift in attitude can be attributed to the increased knowledge acquired through the educational program. As individuals became more informed about the disease and its implications, they developed a more positive attitude towards adopting preventive measures. The findings align with previous studies by Hedayati et al., Zaildo et al., Calcagni et al., and Lapoirie et al., which highlighted the correlation between knowledge acquisition and attitude improvement towards infectious diseases [29–32]. A positive attitude is crucial for promoting compliance with preventive measures and fostering a culture of health and safety within the community.

Furthermore, the intervention resulted in significant changes in self-care behaviors among participants. Individuals in the experimental group exhibited a greater adherence to recommended self-care practices compared to the control group. Through active participation in the WhatsApp group, participants shared their experiences, discussed misconceptions, and received guidance on proper self-care behaviors. This collaborative learning environment facilitated the adoption of effective self-care strategies and promoted a sense of accountability among participants. The study’s findings are consistent with previous research by Yoon et al., Acar et al., Meng et al., Leung et al., Howell et al., Downing et al., Prince et al., and Wu et al., emphasizing the role of education in promoting self-care behaviors related to infectious diseases [33–42].

All in all, the results of this study revealed significant improvements in knowledge, attitude, and self-care behaviors among participants who received the intervention compared to the control group. These findings are consistent with previous research conducted by Nguyen et al., Ryu et al., Byrd et al., Khaday et al., Grano et al.,Navabi et al, Sayed et al., and Elgzar et al. which emphasized the effectiveness of educational interventions in enhancing understanding and preventive behaviors related to infectious diseases [43–48].

### Strengths and limitations

This study’s strengths include the health centers’ participation in its implementation, the intervention’s design based on pre-test results and the use of virtual education techniques, everyone in the group actively participating, daily material presentations, and ample time allotted for participants. The interestingness of the materials supplied to the participants, the capacity to use, save, and keep the materials on a mobile device, and the potential of using them at any time, as well as the continuation of the training after the research is over.

The short-term follow-up of the effect of the implemented training program and the collection of information through questionnaires had limitations, since some of the participants may not have given correct and real information.

## Conclusion

In the present study, the self-learned virtual learning package led to the improvement of self-care behaviors in people who refer to health centers. Considering the importance of the role of health education in promoting self-care behaviors as well as preventing infectious diseases such as COVID-19, it is suggested that educational interventions focus on self-care behaviors in other diseases. In the same way, therefore, it is suggested to pay serious attention to the education and knowledge of people in the community in order to increase the level of knowledge and change their attitude in the implementation of programs to prevent respiratory infections, especially COVID-19 disease.

## Data Availability

No datasets were generated or analysed during the current study.
